# Breaking strength and bone microarchitecture in osteoporosis: a biomechanical approximation based on load tests in 104 human vertebrae from the cervical, thoracic, and lumbar spines of 13 body donors

**DOI:** 10.1186/s13018-022-03105-5

**Published:** 2022-04-11

**Authors:** Guido Schröder, Martin Reichel, Sven Spiegel, Marko Schulze, Andreas Götz, Semjon Bugaichuk, Julian Ramin Andresen, Claus Maximilian Kullen, Reimer Andresen, Hans-Christof Schober

**Affiliations:** 1 Clinic of Orthopedics and Trauma Surgery, Warnow Klinik, 18246 Buetzow, Germany; 2 Faculty of Engineering and Marine Engineering, Chair of Engineering Design/Lightweight Construction, University of Rostock, Rostock, Germany; 3 Medical Faculty, University of Rostock, Rostock, Germany; 4 Institute for Anatomy and Cell Biology, University of Bielefeld, Bielefeld, Germany; 5 Institute for Biomedical Engineering, Medical University of Rostock, Rostock, Germany; 6 University Medical Center Hamburg-Eppendorf (UKE), Hamburg, Germany; 7 Medical School, Sigmund Freud University, Vienna, Austria; 8 Institute of Diagnostic and Interventional Radiology/Neuroradiology, Academic Teaching Hospital of the Universities of Kiel, Luebeck and Hamburg, Westkuestenklinikum Heide, Heide, Germany; 9 Department of Internal Medicine IV, Municipal Hospital Suedstadt Rostock, Academic Teaching Hospital of the University of Rostock, Rostock, Germany

**Keywords:** Osteoporosis, Biomechanics, Computed tomography, Insufficiency fracture, Spine, X-ray microtomography

## Abstract

**Background:**

The purpose of the study was to investigate associations between biomechanical resilience (failure load, failure strength) and the microarchitecture of cancellous bone in the vertebrae of human cadavers with low bone density with or without vertebral fractures (VFx).

**Methods:**

Spines were removed from 13 body donors (approval no. A 2017-0072) and analyzed in regard to bone mineral density (BMD), Hounsfield units (HU), and fracture count (Fx) with the aid of high-resolution CT images. This was followed by the puncture of cancellous bone in the vertebral bodies of C2 to L5 using a Jamshidi™ needle. The following parameters were determined on the micro-CT images: bone volume fraction (BVF), trabecular thickness (Tb.Th), trabecular separation (Tb.Sp), degree of anisotropy (DA), trabecular number (Tb.N), trabecular pattern factor (Tb.Pf), and connectivity density (Conn.D). The axial load behavior of 104 vertebral specimens (C5, C6, T7, T8, T9, T12, L1, L3) was investigated with a servohydraulic testing machine.

**Results:**

Individuals with more than 2 fractures had a significantly lower trabecular pattern factor (Tb.Pf), which also proved to be an important factor for a reduced failure load in the regression analysis with differences between the parts of the spine. The failure load (FL) and endplate sizes of normal vertebrae increased with progression in the craniocaudal direction, while the HU was reduced. Failure strength (FS) was significantly greater in the cervical spine than in the thoracic or lumbar spine (*p* < 0.001), independent of sex. BVF, Tb.Th, Tb.N, and Conn.D were significantly higher in the cervical spine than in the other spinal segments. In contrast, Tb.Sp and Tb.Pf were lowest in the cervical spine. BVF was correlated with FL (*r* = 0.600, *p* = 0.030) and FS (*r* = 0.763, *p* = 0.002). Microarchitectural changes were also detectable in the cervical spine at lower densities.

**Conclusions:**

Due to the unique microarchitecture of the cervical vertebrae, fractures occur much later in this region than they do in the thoracic or lumbar spine.

*Trial registration*

Approval no. A 2017-0072.

## Background

Osteoporosis is a metabolic bone disease in which fractures can occur as a result of even low-energy trauma due to a reduction in bone mass, structure and function [1]. Regardless of sex and age, osteoporotic fractures are found primarily in the area of the distal radius, the proximal femur and the spine [2]. Vertebral fractures (VFx) are associated with health and economic burdens, increased morbidity and mortality, and impaired quality of life [3]. In cases of significantly reduced bone density, VFx occur primarily in the thoracic, thoracolumbar and sacral areas but not in the cervical area [4, 5]. VFx are a strong predictor of future fracture risk independent of bone mineral density (BMD) [6, 7]. The risk of experiencing a new VFx is many times higher in people who have already experienced VFx. A fracture occurs when the force on the vertebra exceeds its strength: Therefore, factors related to both skeletal fragility and spinal loading may play important roles.

The biomechanical competence of vertebrae is determined by the spring-back effect of cortical and cancellous bone [8]. The load-bearing capacity is distributed equally between cancellous and cortical bone in vertebral bodies [9, 10]. With advancing age, the trabecular structure of vertebrae reduces more intensively than that of cortical bone [11].

To the best of our knowledge, no studies of trabecular factors in the microarchitecture range with regard to a VFx event (failure/fracture) in the three sections (cervical, thoracic, lumbar) of the spine have been conducted until now. Therefore, the aim of this work was to examine the trabecular microarchitecture of all three areas of the spine with regard to the systemic course of bone loss. The influence of microstructural changes on the deformation tendency will be investigated. For this purpose, at the beginning of the study a first-degree fracture [12] was created and the failure load was determined particularly in vertebral bodies that frequently fracture in vivo. Second comparisons of people with higher and lower numbers of fractures were carried out.

## Methods

### Design and group assignment

The study was designed as a single center clinical–experimental investigation of an intervention group. Subjects were assigned to groups according to the location of the vertebrae in the individual segments of the spine.

### Recruitment and ethics approval

The probands were recruited from a body donor program at a medical university and had volunteered, during their lifetime, to donate their bodies to scientific research after their death. The investigation was reviewed and approved by the ethics committee (approval no. A 2017-0072).

### Inclusion and exclusion criteria

The criterion for inclusion in the clinical investigation was the presence of 22 vertebral bodies per extracted spine. The exclusion criteria were relevant anatomical deformities: evidence of growth retardation: severe bone diseases such as tumors, bone metastases, Paget’s disease, spinal fusion or the formation of block vertebrae, and previous surgery resulting in foreign material in the spine.

### Extraction and storage of spines

The cadavers were perfused postmortem through the left femoral artery with a 96% ethanol solution at 0.5 bar and stored free-floating in a 0.5% aqueous phenol solution. After the exposure of the superficial (migrated) spinal muscles (the trapezius muscle, rhomboid major and rhomboid minor muscles, levator scapulae muscle, and latissimus dorsi muscle) and the autochthonous back muscles, the ribs were detached paravertebrally by a hand’s breadth and exarticulated at the atlanto-occipital joint, and the ventral vertebral bodies were mobilized bluntly through the retropharyngeal space. At the sacrum, at approximately at the level of the sacroiliac joint, a saw cut was made on both sides and the entire spine was removed from the dorsal aspect. The spines were stored in 70% ethanol at 4 °C until we performed imaging investigations and obtained puncture samples of cancellous bone. The puncture samples were taken from the middle of the vertebral body; the correct position was checked on X-rays.

We obtained samples from 286 previously prepared vertebrae from the ventral medial aspect using a Jamshidi™ needle (8-gauge) and stored the samples in wet towels in a 1.5-ml Eppendorf reaction vessel until further investigation.

### Diagnostic imaging

#### CT and QCT

**Fig. 1:**
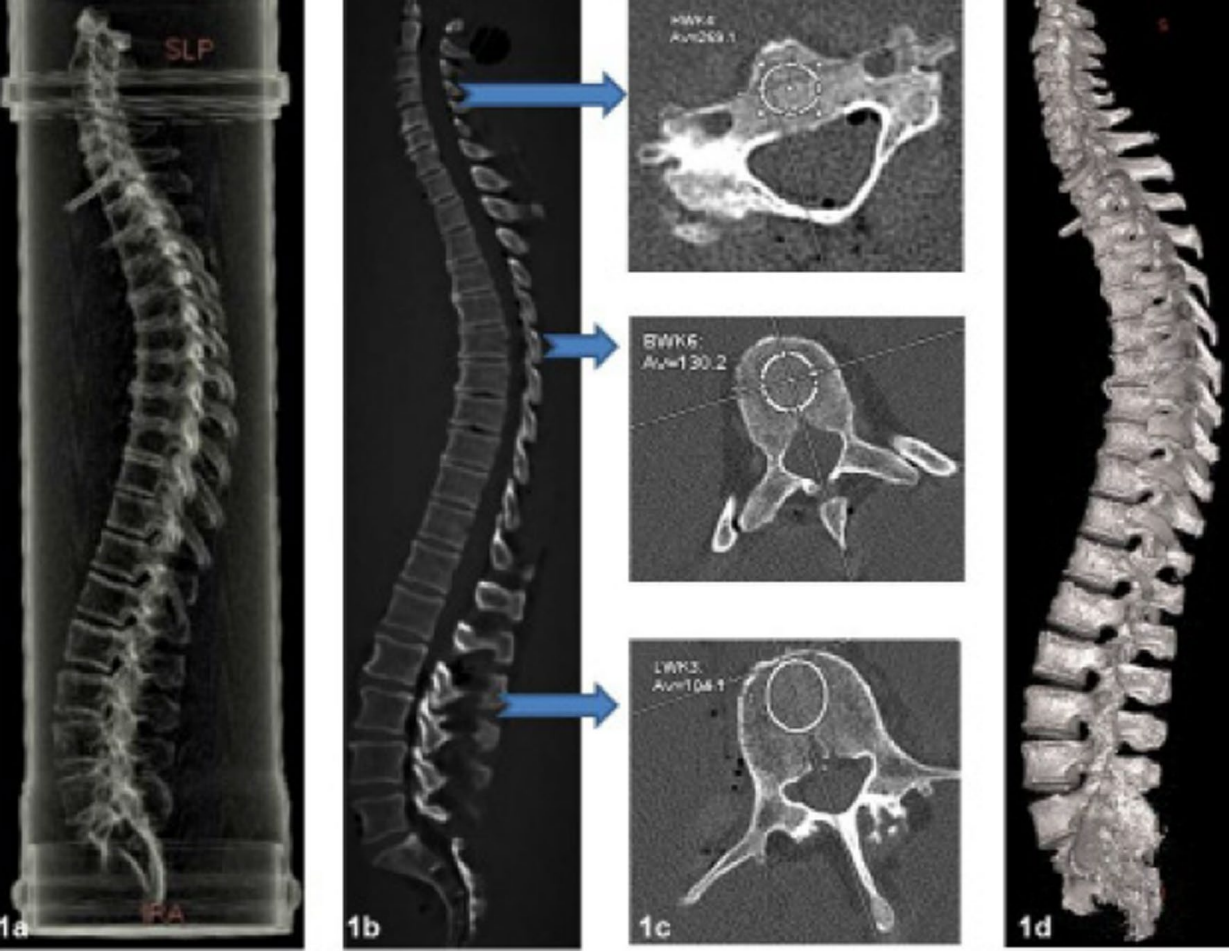
Experimental setup, image reformation, and measurements. **1a** The location of an embedded spine in the PVC tube using a transparent 3D reconstruction. Sagittal reconstructions were obtained from the axial CT scans to detect fractures (**1b**). An ROI was positioned mid-vertebrally in each vertebral body, and the density was determined in HU (**1c**). A 3D reconstruction allowed final assessment of deformities and fractures (**1d**)

To simulate a realistic body size, the donor spines were first fixed in a plexiglass water phantom (Fig. 1a). All spines were then investigated by high-resolution spiral CT (GE Revolution EVO/64 slice CT/lateral scanogram, axial slice thickness < 1 mm, axial and sagittal reconstruction with a slice thickness of 2 mm). Vertebral deformities were identified and graded on sagittal sections (Fig. 1b) [12], and X-ray attenuation was determined in HU by two independent radiologists who also determined the areas of the tested vertebrae. At this time, spines with metastases, any diffuse idiopathic skeletal hyperostosis, or pronounced scoliosis were excluded from further investigation; 13 donor spines were eligible for further evaluation.

To visualize the anatomy of the entire spine, we performed 3D volume rendering at an external workstation (GE AW Server ®, version 2.0., measurement of spines in GE Centricity RIS­i®, Version 5.0) (Fig. 1d). Bone mineral density was measured by QCT (GE Revolution EVO/64-slice computed tomography device and Mindways software, 3D volumetric QCT Spine, Austin, TX, USA). The bone mineral density of cancellous bone was determined in mg/cm^3^ at the level of the lumbar vertebrae L1, L2, and L3; the mean value in mg/cm^3^ was used to estimate the presence of osteoporosis. Additionally, the density of cancellous bone in HU was determined on CT images. These values were obtained for the individual vertebral bodies from the third cervical vertebra (C3) to the fifth lumbar vertebra (L5) (in all 286 vertebrae), using a manually positioned range of interest (ROI) in cancellous bone.

### Micro-CT images and evaluation of microarchitecture

The bone cylinders were investigated using a micro-CT device (SKYSCAN 1172, RJL Micro & Analytic GmbH, Karlsdorf-Neuthart, Germany). We performed flat-field correction and compared the images with phantoms (reference images) with densities of 0.25 g/cm^3^ and 0.75 g/cm^3^. The settings for the scanning process were established as follows: aluminum filter 0.5, resolution 640*512 pixels, pixel size 19.9 µm, isotropic nominal voxel size 35 mm (field of view 70 mm, X-ray source 100 kV, 100 µA).

The trabecular region of interest was defined manually to exclude the cortical component of the vertebra. The following parameters of trabecular microarchitecture were measured: bone volume fraction (BVF, %), trabecular thickness (Tb.Th, µm), trabecular separation (Tb.Sp, µm), degree of anisotropy (DA, 0 = isotropic; 1 = anisotropic), trabecular number (Tb.N, n/mm), trabecular pattern factor (Tb.Pf, mm^−1^), and connectivity density (Conn.D, mm^−3^).

### Mechanical testing

**Fig. 2:**
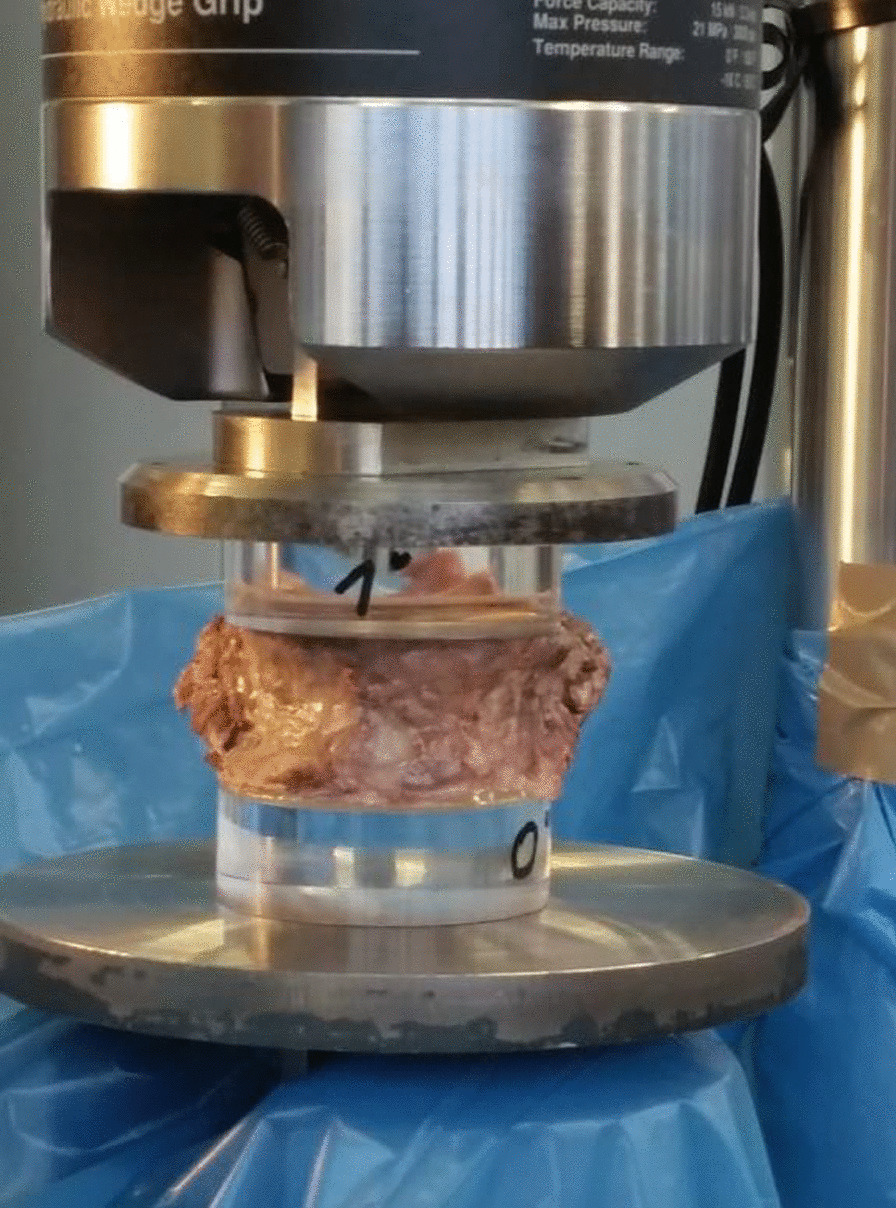
Human vertebral body failure load test setup using an MTS 858 servohydraulic testing machine (MTS Systems Cooperation in Eden Prairie, USA). The testing machine has a nominal force range for static and dynamic tests of ± 15 kilonewtons. The hydraulically driven working piston attached to the crosshead was uniformly moved vertically at the specified test speed of 5 mm/min by opening and closing the control valves. Its counterpart was the load cell located in the base plate. The working piston was moved in the direction of the load cell in the manner of a compressive load on the vertebral body. The force was applied via intervertebral disk replacement material made of an acrylic glass connection. The applied forces and displacements were recorded and stored using the load cell and displacement sensor

The failure loads (N) and failure strengths (N/mm^2^) needed to cause a Grade 1 (20% height reduction) fracture in the load test [6] were determined. Compression of the vertebral bodies (C5, C6, T7, T8, T9, T12, L1, L3) was performed on a servohydraulic testing machine (MTS 858, MTS Systems Cooperation, Eden Prairie, USA, Fig. 2).The hydraulically driven working piston attached to the traverse was moved by opening and closing the control valves vertically in a uniform manner at the prescribed testing speed of 5 mm/min. Force was transmitted through oblique and circular acrylic disks, which served as a substitute for intervertebral disks. Several circular disks were prepared with a band saw for angles of 0° to 10° and left in a rough-sawn state. The applied forces and paths were registered with a force gauge and a displacement sensor; the data were recorded and saved.

### Statistics

All collected data were analyzed with the statistical software package SPSS, version 23.0 (SPSS Inc., Chicago, USA). Quantitative parameters are expressed as means (*M*), standard deviations (SD), and numbers (*n*) of available observations and are presented as the means ± standard deviations (M ± SD). The Kruskal–Wallis test or the one-way analysis of variance (ANOVA) was used for comparisons between groups. Selection was based on the result of the Shapiro–Wilk test for normal distribution. In the case of statistically significant results, we performed pairwise comparisons or the post hoc tests. We used the Mann–Whitney *U* test for the comparison of two groups with non-normally distributed values, and the independent *t* test for the comparison of two groups with normally distributed values. Correlation analyses were performed in accordance with the scale level.

All *p* values are the result of two-sided statistical tests. The level of significance was set to *p* < 0.05.

## Results

A total of 13 spines were retrieved from the body donors and investigated in accordance with the study design. The subjects consisted of four men and nine women aged 73–102 years (mean age, 84.3 ± 8.4 years). Their body height was between 1.47 and 1.79 m (mean height, 1.62 ± 0.11 m) and body weight between 31.1 and 93.7 kg (mean weight, 55.4 ± 16.5 kg). Thus, the mean BMI was 20.7 ± 4.3 kg/m^2^. The available medical records were limited to the cause of death. The subjects' medical history is summarized in Table 1.

**Table 1:** Cases used in this study

	Overall group (*n* = 13)
Age (yr)	84.3 ± 8.4
Sex (male/female)	4/9
Body mass index (kg/m^2^)	20.7 ± 4.3
Bone mineral density (mg/cm^3^)^*^	47.6 ± 24.6
Excluded segments	C3-L5
Vertebral body fractures	2.2 ± 2.0
Total number of vertebrae (n)	286

*Quantitative computed tomography measurements

### QCT

The mean overall bone mineral density of the 13 spines, measured at lumbar vertebrae L1 to L3, was 47.6 ± 24.6 mg/cm^3^, which was indicative of osteoporosis (Table 1). Fractures in the cervical spine were absent. In all of the investigated spines, the density of cancellous bone was significantly higher (*p* = 0.042) in the cervical vertebrae (average177.6 HU) than in the thoracic (average 94.4 HU) or lumbar vertebrae (average 62.8 HU, *p* < 0.001).

**Fig. 3:**
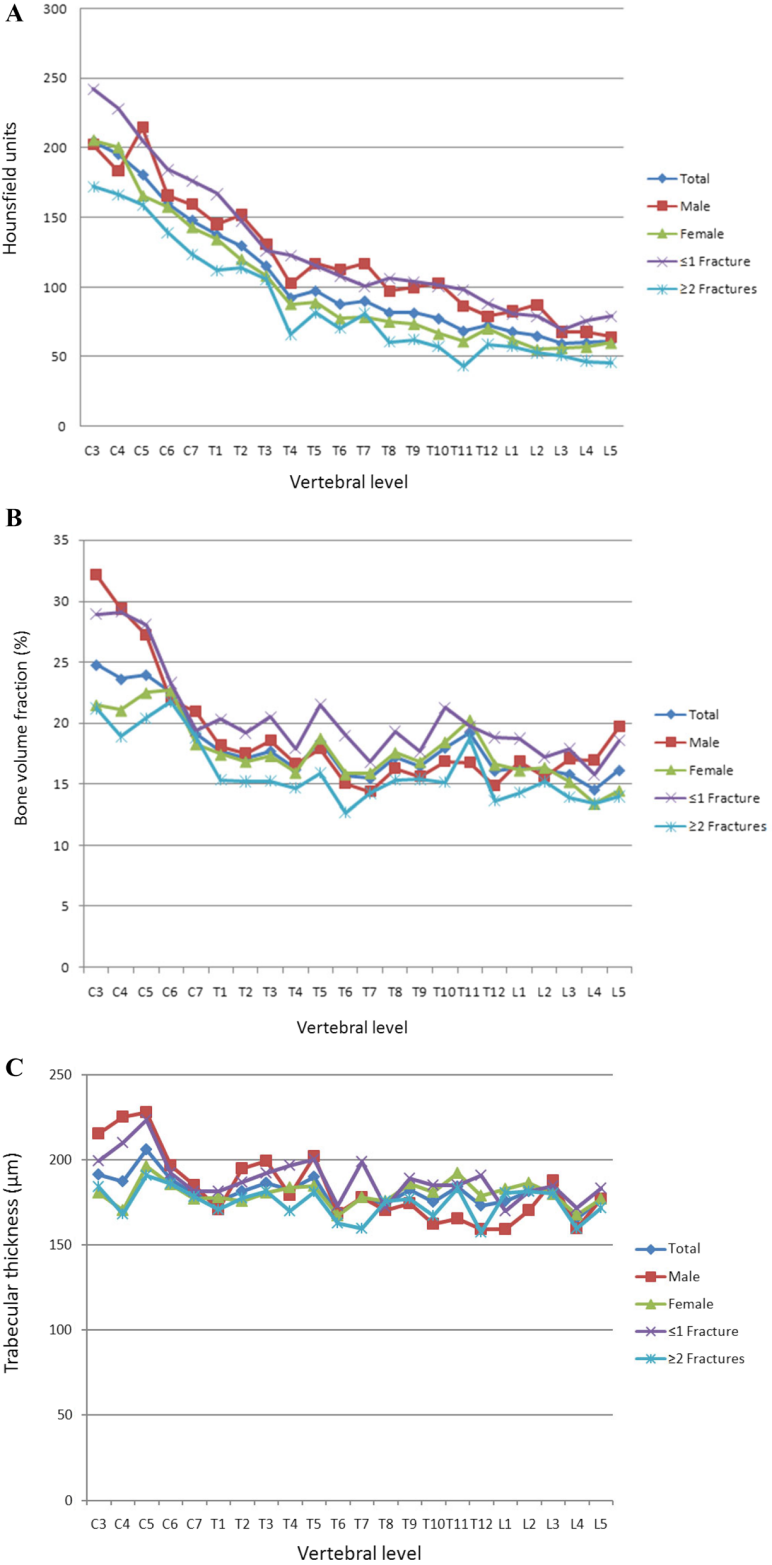

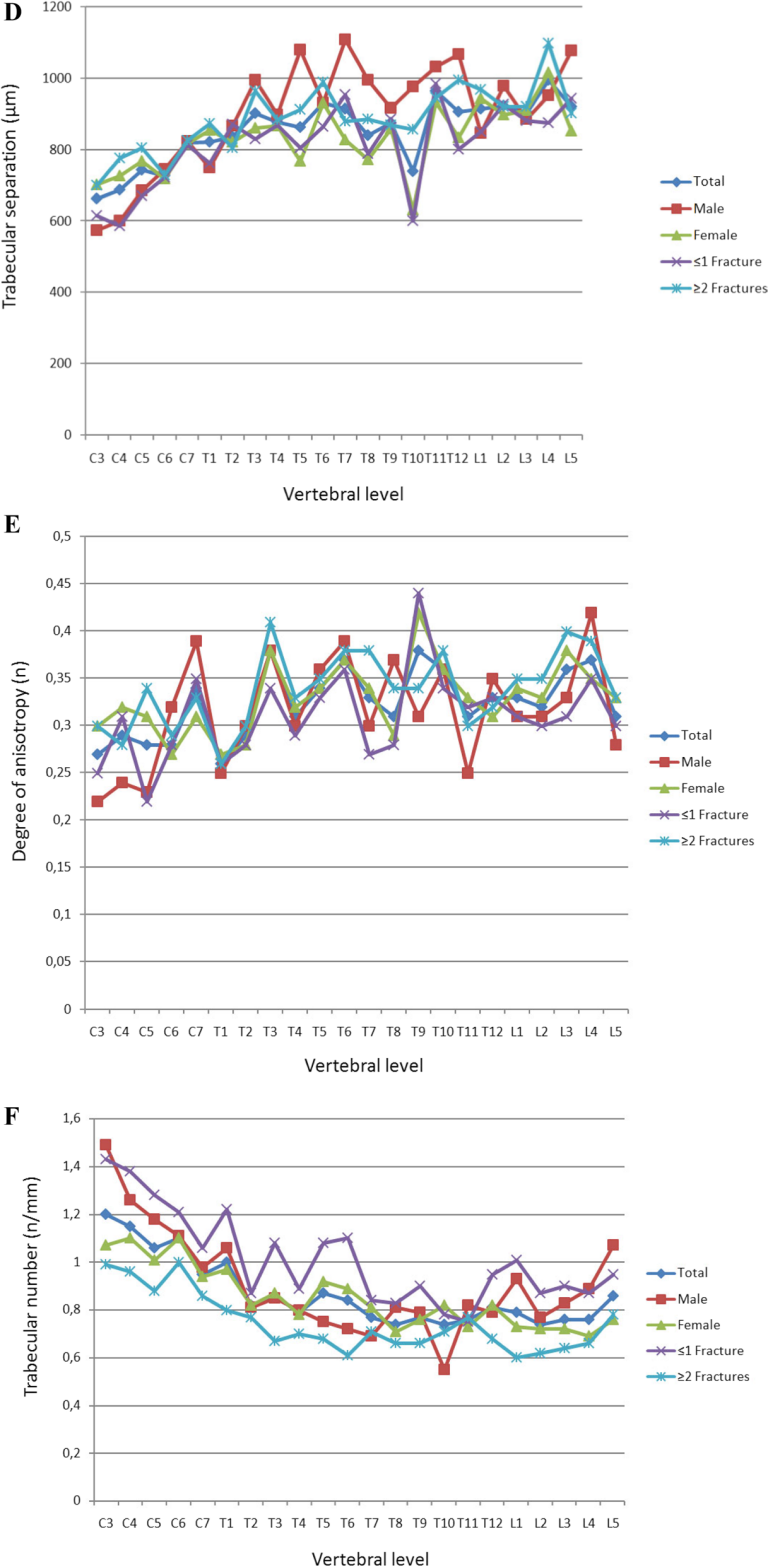

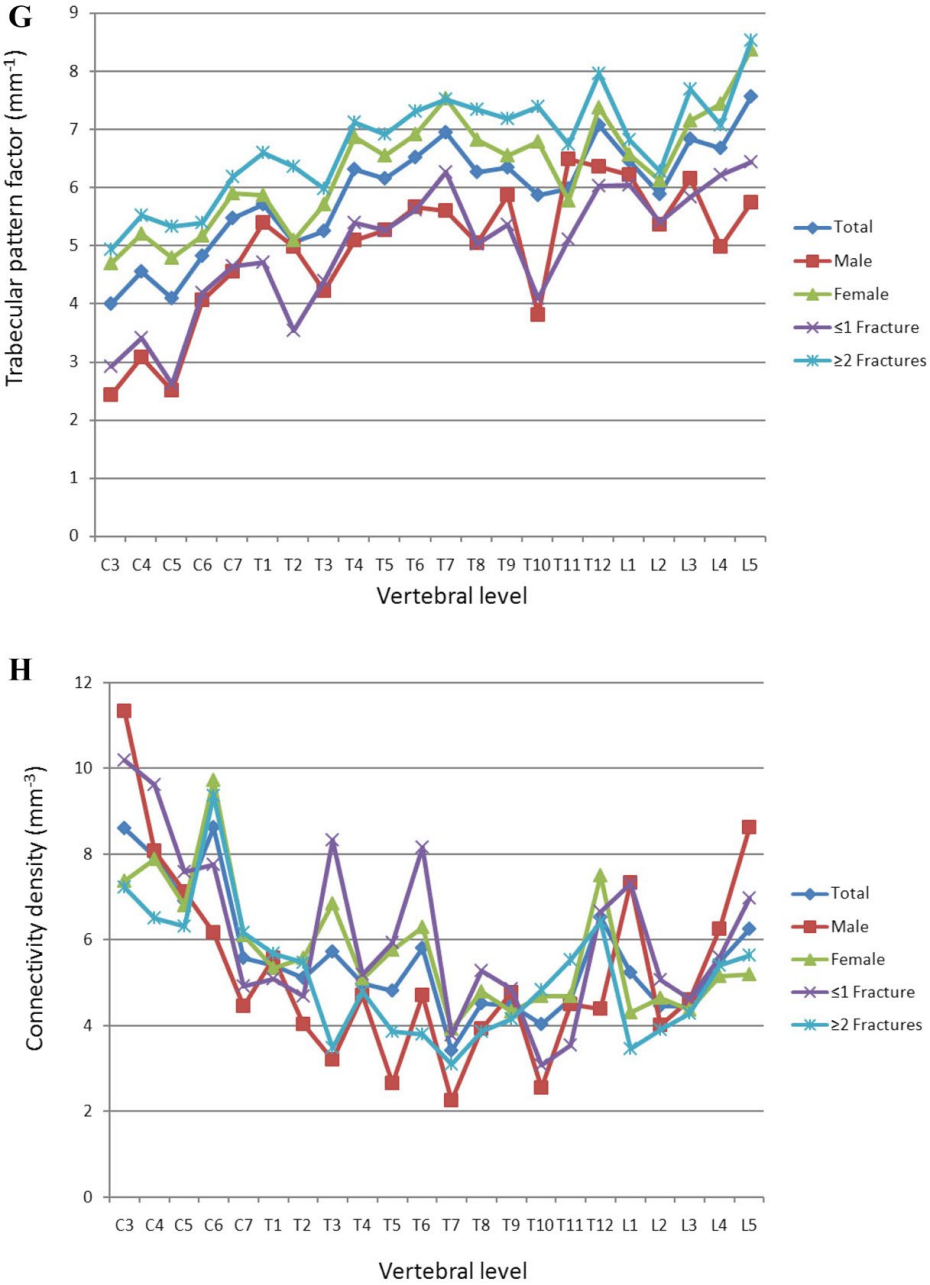
**A** Regional differences in X-ray attenuation of trabecular bone on the Hounsfield scale. Cervical vertebrae had significantly higher HU than thoracic and lumbar vertebrae (Kruskal–Wallis test, *p* values in Table 3). The subgroup analysis using the Mann–Whitney *U* test yielded no significant difference between women and men (*p* = 0.057) but did reveal a significant difference between people with a maximum of one fracture and those with two or more fractures (*p* = 0.005). **B** Regional variations in vertebral trabecular BVF. Cervical vertebrae had a significantly higher BVF than thoracic or lumbar vertebrae (Kruskal–Wallis test, *p* values in Table 2). The subgroup analysis using the Mann–Whitney *U* test revealed no significant difference between women and men but did yield a significant difference between people with a maximum of one fracture and those with two or more fractures (*p* < 0.001). **C** Regional variations in Tb.Th. Cervical vertebrae had a significantly higher Tb.Th more than thoracic or lumbar vertebrae. No significant differences between spinal segments were observed in people older than 80 years of age or in women (ANOVA, post hoc LSD test, *p* values in Table 2). The subgroup analysis using the independent *t* test showed no significant difference between women and men (*p* > 0.05) but did yield a significant difference between people with a maximum of one fracture and those with two or more fractures (*p* < 0.001). **D** Regional variations in Tb.Sp. Cervical vertebrae had a significantly lower Tb.Sp than thoracic or lumbar vertebrae (ANOVA, post hoc LSD test, p values in Table 2). A subgroup analysis using the independent *t* test yielded no significant difference between women and men (*p* > 0.05) but did reveal a significant difference between people with a maximum of one fracture and those with two or more fractures (*p* < 0.025). **E** Regional variations in DA. Cervical vertebrae had a significantly lower DA than thoracic or lumbar vertebrae. In women, no significant differences were observed among the individual spinal segments (ANOVA, post hoc LSD test, *p* values in Table 2). A subgroup analysis using the independent *t* test showed no significant difference between women and men (*p* > 0.05) but did yield a significant difference between people with a maximum of one fracture and those with two or more fractures (*p* < 0.038). **F** Regional variations in Tb.N. Cervical vertebrae had a significantly higher Tb.N than thoracic or lumbar vertebrae (Kruskal–Wallis test, *p* values in Table 2). A subgroup analysis using the Mann–Whitney *U* test yielded no significant difference between women and men (*p* > 0.05) but did reveal a significant difference between people with a maximum of one fracture and those with two or more fractures (*p* < 0.001). **G** Regional variations in Tb.Pf. Cervical vertebrae had a significantly lower Tb.Pf than thoracic or lumbar vertebrae (ANOVA, post hoc LSD test, *p* values in Table 2). The subgroup analysis using the independent *t* test yielded a significant difference between women and men (*p* < 0.001) and between people with a maximum of one fracture and those with two or more fractures (*p* < 0.001). **H** Regional variations in vertebral trabecular Conn.D. Cervical vertebrae had a significantly higher Conn.D than thoracic or lumbar vertebrae (Kruskal–Wallis test, *p* values in Table 2). The subgroup analysis using the Mann–Whitney *U* test yielded no significant difference between women and men or between people with a maximum of one fracture and those with two or more fractures (*p* > 0.05)

The mid-vertebral cancellous bone showed a continuous increase in density with progression toward the cervical vertebrae (Fig. 3A). Comparisons in subgroup analyses also revealed significant differences. The lowest HU was noted in the lumbar spine, independent of sex and previous fractures (Table 3). The mean numbers of vertebral fractures are shown in Table 1. People with a history of more than one vertebral fracture had significantly lower HU values (*p* = 0.005).

### Micro-CT

Differences in the architecture of cancellous bone in relation to the location of the vertebrae are shown in Fig. 3B–H. The lumbar vertebrae had a significantly lower BVF than the cervical vertebrae (Fig. 3B). A pairwise comparison of BVF revealed a significant difference between the cervical and thoracic spine (*p* = 0.023) and a highly significant difference between the cervical and lumbar spine (*p* = 0.001). In contrast, a comparison of the thoracic and lumbar spine yielded no significant difference (*p* > 0.05).

A group comparison of Tb.Th showed a highly significant difference between the cervical and thoracic spine (*p* = 0.008) and between the cervical and lumbar spine (*p* = 0.006). The difference between the thoracic and lumbar spine, on the other hand, was not significant (*p* > 0.05). The closest proximity of trabeculae was observed in the cervical spine (Fig. 3D). Comparison of the cervical and thoracic spine and of the cervical and lumbar spine yielded highly significant differences (*p* < 0.001). A comparison of the thoracic and lumbar spine revealed no significant difference (*p* > 0.05). The degree of anisotropy was lowest in the cervical spine (Fig. 3E). Group comparisons showed no significant difference between the various segments of the spine (*p* > 0.05).

The number of trabecular was highest in the cervical spine (Fig. 3F). Significantly more trabeculae were found in the cervical vertebrae than in the thoracic or lumbar vertebrae (*p* = 0.005) (Fig. 3F). A pairwise comparison of values yielded a significant difference between the cervical and thoracic spine (*p* = 0.014) and a highly significant difference between the cervical and lumbar spine (*p* = 0.005). In contrast, no significant difference was noted between the thoracic and lumbar spine (*p* > 0.05).

In general, the trabecular pattern factor was lowest in the cervical spine (Fig. 3G). Comparison of values between the cervical and thoracic spine and between the cervical and lumbar spine yielded a highly significant difference (*p* < 0.001), whereas the values of the thoracic and lumbar spine did not differ significantly (*p* > 0.05). The trabecular network was denser in the cervical spine than in the thoracic (*p* < 0.001) and lumbar spine (*p* = 0.001). In contrast, the difference between the thoracic and lumbar spine was not significant (*p* > 0.05). The data are summarized in Table 2.

**Table 2:** Descriptive statistics for microcomputed tomography parameters

				Spinal section				Group comparison		
Architectural parameter	Group	Total		CS	TS	LS		CS vs. TS *p* value	CS vs. LS *p* value	TS vs. LS *p* value
BVF (%)	Total	18.09 ± 2.97		22.79 ± 2.22	17.09 ± 1.17	15.79 ± 0.74		0.023^P^	0.001^P^	0.401^P^
	Male	18.95 ± 4.76		26.33 ± 4.81	16.57 ± 1.34	17.27 ± 1.50		0.002^P^	0.085^P^	1.000^P^
	Female	17.71 ± 2.53		21.22 ± 1.77	17.33 ± 1.32	15.13 ± 1.22		0.055^P^	0.001^P^	0.124^P^
	≤ 1Fx	20.43 ± 3.75		25.76 ± 4.28	19.36 ± 1.43	17.66 ± 1.20		0.070^P^	0.002^P^	0.196^P^
	≥ 2Fx	16.09 ± 2.64		20.24 ± 1.32	15.15 ± 1.43	14.19 ± 0.67		0.015^P^	0.002^P^	0.569^P^
Tb.Th (µm)	Total	182 ± 9		191 ± 10	180 ± 6	177 ± 7		0.008^L^	0.006^L^	0.477^L^
	Male	184 ± 21		211 ± 19	178 ± 14	171 ± 12		0.001^L^	0.001^L^	0.446^L^
	Female	181 ± 7		182 ± 10	181 ± 6	179 ± 7				
	≤ 1 Fx	189 ± 13		202 ± 16	188 ± 9	179 ± 7		0.026^L^	0.003^L^	0.116^L^
	≥ 2 Fx	175 ± 9		182 ± 9	172 ± 9	175 ± 9				
Tb.Sp (µm)	Total	854 ± 91		729 ± 60	874 ± 59	933 ± 37		< 0.001^L^	< 0.001^L^	0.060^L^
	Male	901 ± 152		687 ± 103	969 ± 102	950 ± 90		< 0.001^L^	0.001^L^	0.717^L^
	Female	834 ± 91		748 ± 47	831 ± 81	925 ± 61		0.038^L^	0.001^L^	0.022^L^
	≤ 1 Fx	815 ± 114		683 ± 90	836 ± 99	897 ± 38		0.004^L^	0.001^L^	0.206^L^
	≥ 2 Fx	888 ± 92		768 ± 52	906 ± 58	963 ± 80		0.001^L^	< 0.001^L^	0.098^L^
DA (n)	Total	0.33 ± 0.04		0.29 ± 0.03	0.33 ± 0.04	0.34 ± 0.03				
	Male	0.32 ± 0.06		0.28 ± 0.07	0.33 ± 0.05	0.33 ± 0.05				
	Female	0.33 ± 0.04		0.30 ± 0.02	0.33 ± 0.04	0.35 ± 0.02				
	≤ 1 Fx	0.31 ± 0.05		0.28 ± 0.05	0.32 ± 0.05	0.31 ± 0.02				
	≥ 2 Fx	0.34 ± 0.04		0.31 ± 0.03	0.34 ± 0.04	0.36 ± 0.03				
Tb.N (n/mm)	Total	0.87 ± 0.14		1.09 ± 0.09	0.81 ± 0.07	0.78 ± 0.05		0.014^P^	0.005^P^	1.000^P^
	Male	0.91 ± 0.21		1.20 ± 0.19	0.79 ± 0.12	0.90 ± 0.12		0.002^P^	0.293^P^	0.394^P^
	Female	0.85 ± 0.13		1.04 ± 0.07	0.83 ± 0.08	0.73 ± 0.02		0.037^P^	< 0.001^P^	0.114^P^
	≤ 1 Fx	1.01 ± 0.19		1.27 ± 0.15	0.94 ± 0.15	0.92 ± 0.06		0.015^P^	0.051^P^	1.000^P^
	≥ 2 Fx	0.75 ± 0.12		0.94 ± 0.07	0.70 ± 0.05	0.66 ± 0.07		0.015^P^	0.002^P^	0.569^P^
Tb.Pf (mm^−1^)	Total	5.91 ± 0.96		4.59 ± 0.60	6.13 ± 0.60	6.69 ± 0.61		< 0.001^L^	< 0.001^L^	0.097^L^
	Male	4.95 ± 1.17		3.32 ± 0.94	5.32 ± 0.78	5.69 ± 0.53		< 0.001^L^	< 0.001^L^	0.373^L^
	Female	6.33 ± 0.98		5.16 ± 0.47	6.49 ± 0.73	7.13 ± 0.86		0.002^L^	< 0.001^L^	0.109^L^
	≤ 1 Fx	4.94 ± 1.10		3.56 ± 0.85	5.07 ± 0.78	5.99 ± 0.39		0.001^L^	< 0.001^L^	0.030^L^
	≥ 2 Fx	6.74 ± 0.92		5.48 ± 0.45	7.04 ± 0.55	7.29 ± 0.86		< 0.001^L^	< 0.001^L^	0.449^L^
Conn.D (mm^−3^)	Total	5.60 ± 1.41		7.55 ± 1.29	4.96 ± 0.85	5.19 ± 0.76		< 0.001^P^	0.001^P^	0.662^P^
	Male	5.25 ± 2.20		7.44 ± 2.56	3.95 ± 1.05	6.18 ± 1.90		0.001^P^	0.244^P^	0.021^P^
	Female	5.76 ± 1.45		7.59 ± 1.38	5.42 ± 1.06	4.75 ± 0.42		0.001^P^	< 0.001^P^	0.243^P^
	≤ 1 Fx	6.12 ± 1.93		8.03 ± 2.07	5.40 ± 1.67	5.93 ± 1.17		0.008^P^	0.061^P^	0.558^P^
	≥ 2 Fx	5.16 ± 1.50		7.13 ± 1.32	4.59 ± 1.03	4.55 ± 0.94		< 0.001^P^	0.001^P^	0.943^P^

Values are presented as means ± SD. CS, cervical spine; TS, thoracic spine; LS, lumbar spine; BVF, bone volume fraction; Tb.Th, trabecular bone thickness; Tb.Sp, trabecular separation; DA, degree of anisotropy; Tb.N, trabecular number; Tb.Pf, trabecular pattern factor; Conn.D, connectivity density; Fx, fracture; ^P^, pairwise comparison; and ^L^, post hoc LSD test; in the absence of *F* test significance, no pairwise comparisons were tested

### Subgroup analysis

In a subgroup analysis, we viewed the subjects' sex and number of previous fractures independent of each other. Regardless of sex and fracture count, the QCT of the cervical spine had the highest HU value (Table 3). The HU values were significantly lower in the group with ≥ 2 fractures (*p* = 0.005) than in the group with ≤ 1fracture. Regarding HU, a statistical trend was noted between men and women (*p* = 0.057).

**Table 3:** Descriptive statistics for computed tomography parameters

			Spinal section			Group comparison		
Architectural parameter	Group	Total	CS	TS	LS	CS vs. TS *p* value	CS vs. LS *p* value	TS vs. LS* p* value
Hounsfield units	Total	106.1 ± 45.9	177.6 ± 23.6	94.4 ± 22.0	62.8 ± 3.6	0.042^P^	< 0.001^P^	0.042^P^
	Male	119.9 ± 44.0	185.2 ± 23.5	111.8 ± 22.2	74.0 ± 10.3	0.034^P^	< 0.001^P^	0.081^P^
	Female	100.0 ± 47.4	174.2 ± 27.4	86.6 ± 22.6	57.9 ± 2.7	0.039^P^	< 0.001^P^	0.052^P^
	≤ 1 Fx	127.7 ± 51.3	207.3 ± 28.1	115.6 ± 22.6	76.9 ± 4.6	0.042^P^	< 0.001^P^	0.042^P^
	≥ 2 Fx	87.6 ± 41.9	152.1 ± 20.3	76.1 ± 23.3	50.7 ± 4.6	0.030^P^	< 0.001^P^	0.121^P^

Values are presented as means ± SD; CS, cervical spine; TS, thoracic spine; LS, lumbar spine; Fx, fracture; and ^P^, pairwise comparison

The micro-CT parameters revealed a significantly higher BVF in the cervical spine than in the thoracic or lumbar spine in people with ≥ 2 fractures (Table 2, Fig. 3B). This group had a significantly lower BVF (*p* < 0.001) than the group with ≤ 1 fracture. Men and people with a history of ≤ 1 fracture had a significantly higher trabecular density in the cervical spine than in the thoracic or lumbar spine (Table 2, Fig. 3C). Regarding this parameter, no significant difference was observed between the individual segments of the spine in women and in people with ≥ 2 fractures (*p* > 0.05). The latter group had significantly thinner trabeculae (*p* < 0.001, Fig. 3C) than the group with ≤ 1 fracture.

Tb.Sp is known to increase from the cervical to caudal direction, regardless of sex and across all fracture counts (Table 2). A significant difference was noted between people with ≤ 1fracture and those with ≥ 2 fractures (*p* = 0.025, Fig. 3D). DA did not differ significantly among the individual segments of the spine (Table 2). People with ≥ 2 fractures had a significantly higher DA than those with ≤ 1 fracture (*p* = 0.038, Fig. 3E). No significant difference in this measurement was noted between men and women (*p* > 0.05).

The number of trabecular was highest in the cervical spine, independent of sex and fracture count (Table 2). In contrast, the Tb.Pf was lower in the cervical spine than in the thoracic or lumbar spine, independent of sex and fracture count (Table 2). With regard to this parameter, there was a highly significant difference between men and women and between the group with ≤ 1 fracture and the group with ≥ 2 fracture (*p* < 0.001, Fig. 3G).

Conn.D was highest in the cervical spine, independent of sex and fracture count (Table 2). There was no significant difference in this parameter between men and women or between people with ≤ 1fracture and those with ≥ 2 fractures (*p* > 0.05, Fig. 3H) in this regard.

### Failure loads and failure strengths

**Fig. 4:**
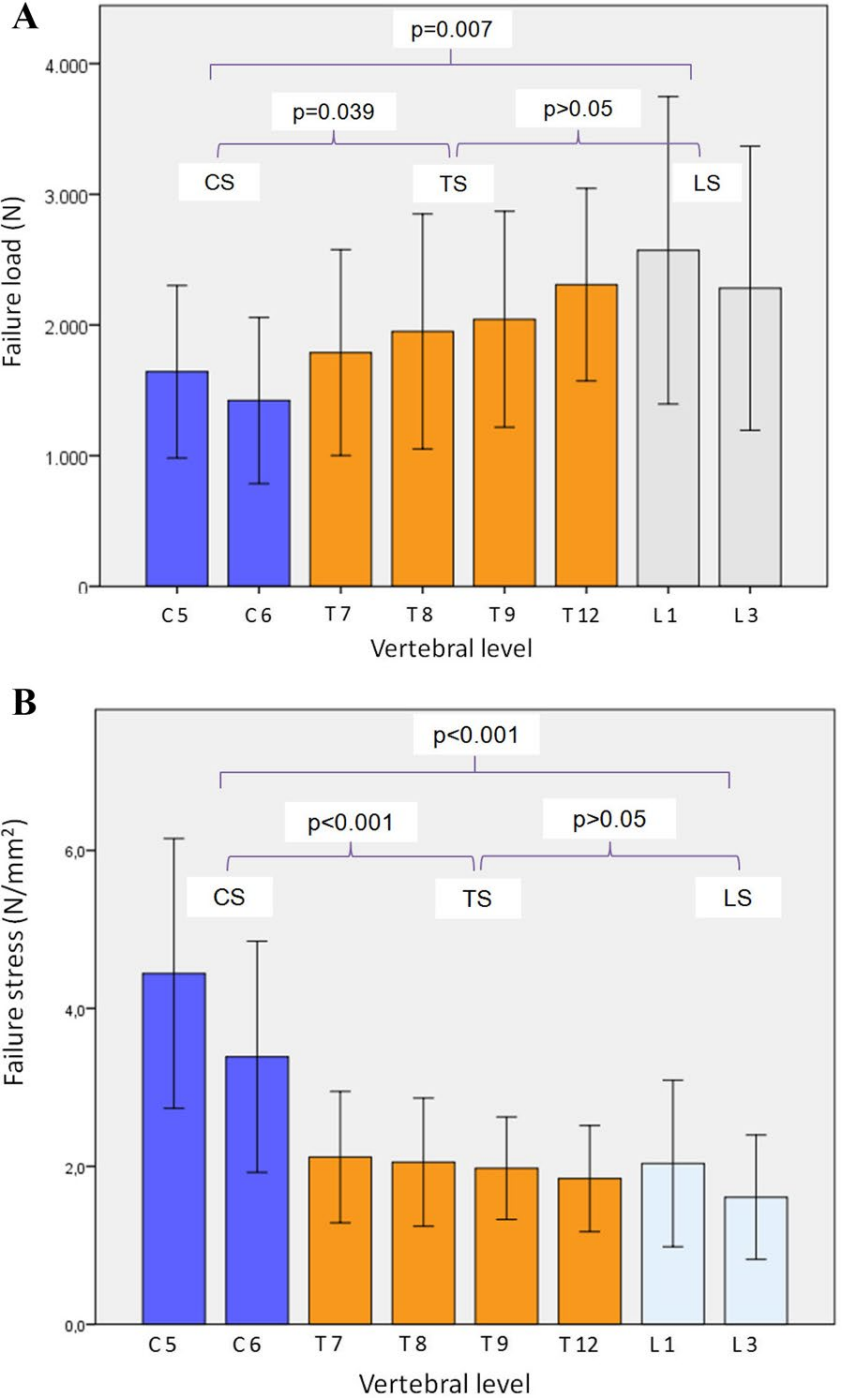
**A** Regional variations in vertebral body failure load; CS, cervical spine; TS, thoracic spine; and LS, lumbar spine. Cervical vertebrae fracture had a significantly lower failure load than thoracic or lumbar vertebrae. **B** Regional variations in vertebral body failure strength; CS, cervical spine; TS, thoracic spine; LS, lumbar spine. In terms of area fractions, cervical vertebrae had a significantly higher failure strength than thoracic and lumbar vertebrae

The failure loads required to cause a Grade I fracture are shown in Table 4. The failure loads increased as with progression in the craniocaudal direction. A statistically significant difference was noted between the cervical and thoracic spine (*p *= 0.039) and between the cervical and lumbar spine (*p* = 0.007). In contrast, no difference was observed between the thoracic and lumbar spine (*p* > 0.05, Fig. 4A). Regarding FL, we found a significant difference between men and women (*p* = 0.015). The thoracic vertebrae T7 (*p* = 0.020), T8 (*p* = 0.018) and T9 (*p* = 0.023) were particularly in this regard. In contrast, a comparison of the groups with ≤ 1 fracture versus ≥ 2 fractures yielded a significant differences for the cervical vertebra C5 (*p* = 0.033) and the thoracic vertebra T8 (*p* = 0.05).

**Table 4:** Failure load and failure strength in relation to gender and fracture numbers

Parameters	Total (*n* = 13) M ± SD	Female (*n* = 9) M ± SD	Male (*n* = 4) M ± SD	≤ 1 Fx M ± SD	≥ 2 Fx M ± SD	Group comparison *p* value*p value**	
Failure load (*N*) Total	2001 ± 650	1728 ± 543	2616 ± 421	2301 ± 385	1744 ± 744	0.015	0.127
C5	1642 ± 661	1457 ± 517	2059 ± 836	2049 ± 742	1293 ± 328	0.135	0.033
C6	1422 ± 635	1198 ± 458	1928 ± 751	1713 ± 725	1173 ± 461	0.051	0.131
T7	1789 ± 788	1469 ± 477	2507 ± 937	2094 ± 453	1527 ± 946	0.020	0.208
T8	1951 ± 899	1580 ± 695	2785 ± 779	2466 ± 568	1509 ± 925	0.018	0.05
T9	2044 ± 826	1714 ± 736	2784 ± 478	2407 ± 451	1732 ± 975	0.023	0.149
T12	2308 ± 737	2080 ± 746	2823 ± 422	2662 ± 485	2005 ± 810	0.093	0.111
L1	2572 ± 1176	2358 ± 1150	3051 ± 1250	2698 ± 940	2463 ± 1414	0.349	0.736
L3	2282 ± 1087	1967 ± 1137	2990 ± 563	2321 ± 1049	2249 ± 1202	0.121	0.910
Failure strength (N/mm^2^)Total	2.4 ± 0.8	2.3 ± 0.8	2.8 ± 0.7	2.7 ± 0.5	2.2 ± 0.9	0.267	0.189
C5	4.4 ± 1.7	4.1 ± 1.5	5.3 ± 2.0	5.4 ± 1.9	3.6 ± 0.9	0.265	0.068
C6	3.4 ± 1.5	3.0 ± 1.3	4.2 ± 1.6	3.8 ± 1.5	3.0 ± 1.5	0.198	0.313
T7	2.1 ± 0.8	1.9 ± 0.8	2.5 ± 1.0	2.3 ± 0.6	1.9 ± 1.0	0.240	0.386
T8	2.1 ± 0.8	1.8 ± 0.8	2.5 ± 0.8	2.5 ± 0.6	1.7 ± 0.8	0.151	0.047
T9	2.0 ± 0.6	1.8 ± 0.7	2.3 ± 0.3	2.3 ± 0.2	1.7 ± 0.8	0.224	0.161
T12	1.8 ± 0.7	1.8 ± 0.8	1.9 ± 0.3	2.0 ± 0.4	1.7 ± 0.9	0.964	0.554
L1	2.0 ± 1.1	2.1 ± 1.2	2.0 ± 0.9	2.0 ± 0.9	2.1 ± 1.3	0.878	0.932
L3	1.6 ± 0.8	1.5 ± 0.9	1.8 ± 0.3	1.5 ± 0.6	1.7 ± 1.0	0.652	0.710

Values are presented as means ± SD; *group comparison male vs. female, ** group comparison ≤ 1 fracture (Fx) vs. ≥ 2 fractures, independent *t* test

There was a highly significant difference in failure strengths between the cervical and thoracic spine and between the cervical and lumbar spine (*p* < 0.001). In contrast, no significant difference was noted between the thoracic and lumbar spine (*p* > 0.05, Fig. 4B). A closer look at the individual vertebrae revealed no sex-specific differences (*p* > 0.05). In contrast, a comparison of people with ≤ 1 fracture and those with ≥ 2 fractures yielded a significant difference for the thoracic vertebra T8 (*p* = 0.047) and a statistical trend for the cervical vertebra C5 (*p* = 0.068, Table 4).

The correlation of micro-CT parameters in the tested vertebral bodies with the determined failure loads yielded significant results for BVF (*r* = 0.67, *p* = 0.012), Tb.Th (*r* = 0.58, *p* = 0.037), and Tb.Pf (*r* = − 0.81, *p* = 0.001) (Table 5). Regarding failure strength, statistically significant correlations were registered for BVF (*r* = 0.76, *p* = 0.002), Tb.Th (*r* = 0.83, *p* < 0.001), DA (*r* = − 0.64, *p* = 0.018), and Tb.Pf (*r* = − 0.79, *p* = 0.001).

**Table 5:** Pearson’s correlation coefficients between bone mass parameters, trabecular microarchitecture parameters, and mechanical behavior

	BMD	HU	BVF	Tb.Th	Tb.Sp	DA	Tb.N	Tb.Pf	Conn.D	Failure load	Failure stress
BMD (mg/cm^3^)											
HU	0.74**										
BVF (%)	0.76**	0.86**									
Tb.Th (µm)	0.55	0.81**	0.82**								
Tb.Sp (µm)	− 0.23	− 0.05	− 0.34	− 0.26							
DA (n)	− 0.19	− 0.67	− 0.54	− 0.61*	− 0.30						
Tb.N (n/mm)	0.60*	0.48	0.71**	0.51	− 0.85**	− 0.01					
Tb.Pf(mm^−1^)	− 0.62*	− 0.84**	− 0.85**	− 0.67*	0.09	0.71**	− 0.54				
Conn.D(mm^−3^)	0.43	0.52	0.66*	0.49	− 0.39	− 0.37	0.46	− 0.41			
Failure load (N)	0.52	0.70**	0.67*	0.58*	− 0.04	− 0.51	0.46	− 0.81**	0.05		
Failure strength (N/mm^2^)	0.56*	0.86**	0.76**	0.83**	− 0.09	− 0.64*	0.49	− 0.79**	0.19	0.84**	

BMD, bone mineral density; HU, Hounsfield units; BVF, bone volume fraction; Tb.Th, trabecular thickness; Tb.Sp, trabecular separation, DA, degree of anisotropy; Tb.N, trabecular number; Tb.Pf, trabecular pattern factor; and Conn.D, connectivity density

^*^p < 0.05; **p < 0.01; and ***p < 0.001

Furthermore, significant and highly significant correlations were noted between HU and failure load (*r* = 0.70, *p* = 0.008) and between HU and failure strength (*r* = 0.86, *p* < 0.001). A significant correlation was also noted between the determined lumbar bone density and failure strength (*r* = 0.56, *p* = 0.048).

### Combined effects of trabecular bone on mechanical behavior

Stepwise regression analyses were performed to determine the effect of the trabecular microarchitecture on the failure load and failure strength. To explain failure loads, we first examined the micro-CT parameters with the highest correlations (BVF, Tb.Th, Tb.Pf). Tb.Pf was found to be the best predictor of failure load (*R*^2^ = 0.62, *p* = 0.001) (Table 6). To explain failure strength, we first addressed the parameters BVF, Tb.Th, DA, and Tb.Pf. The combination of Tb.Th and Tb.Pf proved to be the best predictor of stiffness on bending (*R*^2^ = 0.75, *p* < 0.001) (Table 6).

**Table 6:** Multiple regression analysis including the coefficient of determination (*R*^2^), and the *p* value, for each variable included in the models

Dependent	Independent	Final *R*^2^	*p* value
Variables			
Failure load	Tb.Pf	0.62	0.001
Failure strength	Tb.Pf Tb.Th	0.75	0.049 0.019 < 0.001

## Discussion

### Discussion of results

The present investigation permitted a comparison of the trabecular bone in all segments of the spines of 13 body donors aged 73–102 years. All investigated probands had osteoporosis according to bone densitometry; vertebral fractures were observed in those with the lowest bone densities.

Notably, significantly higher bone density was observed in the trabecular structure of the cervical spine than in that of the thoracic or lumbar spine. Grote et al. [13] found in a histomorphometric study that the density of trabecular bone in the cervical spine is markedly higher than in the thoracic or lumbar spine. They showed that the loss of bone mass with age is lowest in the cervical spine and observed no significant age-related loss of trabecular density in the cervical vertebrae C3 and C4; this concurs with the outcome of our investigation. Schröder et al. [4, 5] reported similar data in their case report and in a preliminary pilot study.

In the present investigation, the bone volume fraction (BVF) differed significantly in the individual segments of the spine; the highest values were noted in the cervical spine. Knowledge of regional differences in microstructure is crucial for the assessment of age- and sex-related bone loss in vertebrae and provides greater insights into the pathomechanisms of spinal osteoporosis and the accompanying risk of fractures [14]. Fractures are associated with age-related trabecular bone microdamage, which is caused partly by the reduction of BVF [15]. BVF, in turn, is determined by Tb.N and Tb.Sp. The reduction in Tb.N with age causes an increase in Tb.Sp. [14].

In our investigation, the cervical vertebrae revealed more numerous and thicker trabeculae, located in significantly greater proximity to one another. This is indicative of the superior biomechanical competence of the cervical vertebrae. Biomechanical competence is also reflected in studies of failure strength. The tested cervical vertebrae were able to absorb significantly more force per unit area than the thoracic and lumbar vertebrae. In addition, cervical vertebrae are smaller than other vertebrae, probably because of the reduced load requirements in this region. However, due to their anatomical structure, cervical vertebrae offer more numerous options than other types of vertebrae for muscle insertions; this may be the reason for the higher mechanical loads in this region. Our results also show that people with more than 2 VFx also have reduced micro-CT parameters in the cervical spine. These results again show that osteoporosis is a systemic process. In general, the vertical struts in vertebral trabecular bone have a thickness of 100–200 µm, whereas the Tb.Sp. of vertical trabeculae is approximately 600–900 µm [16–18]. Our values are in agreement with published data. However, any analysis of trabecular bone structure must distinguish between the vertical and horizontal components of trabecular bone.

In our study, the degree of anisotropy (DA) did not differ significantly in the individual segments of the spine. A more anisotropic structure is caused by the deterioration of trabecular bone architecture in vertebrae, which in turn signifies a greater susceptibility to fractures [17].

The load-bearing capacity of vertebrae increases in direct proportion to their size [19]. Due to their position alone, lumbar vertebrae are subject to greater loads because the body weight acting on the vertebrae increases with progression in the caudal direction [20].

The different loads of the individual spinal segments are reflected by their different fracture rates. Fractures occur primarily in the mid-thoracic spine (T7, T8) and at the junction of the thoracic and lumbar spine (T12 to L1) [21]. One explanation for this fracture cascade along the spine could be the curvatures of the spine [22]. The turning point of the curvature in thoracic kyphosis is at the mid-portion of the thoracic spine (T7, T8) because of the resulting torque force.

The microarchitecture is also of importance. Our stepwise and multiple regression analyses revealed that Tb.Pf was the best predictor of failure load, whereas the combination of Tb.Th and Tb.Pf was the best predictor of failure strength. In general, our results indicate that in addition to the thickness of individual trabeculae, their connectivity density influences their load-bearing capacity. Roux et al. [23] established the combination of BMD, SMI, and Tb.Th as the best predictor of failure load and found the combination of BMD, Tb.Th and the curvature of the anterior cortical bone was the best predictor of failure strength. Roux et al. [23] investigated only lumbar spines, whereas we studied vertebrae from all spinal segments especially the vertebrae that are prone to fractures. The effect of bone loss was obvious in people with 2 or more fractures. Their microarchitectural changes (Fig. 3B–G) were significantly greater than those of people with one or no fracture. These results explain the lower failure strength in people with 2 or more fractures and raise the possibility that there is a threshold for fracturing.

The load-bearing capacity of a spinal segment depends on the material properties of the vertebrae in that region and their geometrical dimensions. Since the material properties of bone tissue are largely predetermined, the dimensions of vertebrae may be viewed as a result of their adjustment to loads acting upon them from the outside and the inside (body weight, muscle activity, preload of ligaments, applied external force) [24].

Our investigation showed that parameters of bone mass (HU) are strongly correlated with the failure strength and failure loads of vertebrae. Some, but not all features of the trabecular microarchitecture were correlated with mechanical behavior. Fractures may occur because bones become too flexible or too weak, do not absorb sufficient energy, and/or are not resistant to repetitive loads.

Bone density, as an important factor of strength, is determined using various methods: However, it can be simply specified using standardized CT-Hounsfield units (HU). Above all, the assessment of bone density becomes increasingly important as patients age. Determining bone quality is crucial for the success of treatment, especially preventive treatment for osteoporotic fractures, but it is also part of optimal surgical preparation for spinal surgery [25]. Dual-energy X-ray absorptiometry (DEXA) is considered the gold standard for determining bone density and identifying osteoporosis [26]: However, this technology is not universally available, and assessment using HU with standard CT can provide a reliable estimate of bone density, improving diagnostic performance and reducing unnecessary radiation exposure [25].

The workload, muscle strength, and load-bearing capacity of the cervical vertebrae are entirely different from those of the thoracic and lumbar vertebra. The lower vertebrae are naturally more prone to attrition and wear than cervical vertebrae. This fact must be considered in any discussion of the subject.

However, the spine is not a rigid entity. It is loaded in dynamic rather than static fashion, not by individual forces or momentum but by combined dynamic loads (such as flexion, compression, torsion, and shearing) in various spatial directions. The human motion segments react to dynamic loads in a nonlinear viscoelastic fashion that can be described as a process of hysteresis [27].Thus, a small deflection requires a relatively small force, whereas greater deflections result in elastic restoring force due to the complexity of the existing system of ligaments and muscles [24, 28]. Thus, it may be assumed that adjustments to the microarchitecture of the cervical vertebrae occur because of their immense mobility. Furthermore, the position of the momentary rotation axis in the cervical spine is prone to constant change, which in turn determines the reaction of a vertebra to the loads acting upon it at any given time [24].

Increased curvature is responsible for higher bending moments and compression forces. This theory is supported by the fact that the radius of curvature increases during life, which signifies greater kyphosis. This is especially significant in osteoporosis and results in greater bending loads. Similarly, the higher risk of fractures at the junction of T12/L1 could be explained by the greater mobility of the lumbar spine and the resulting increase in compressive loads.

In addition to the individual shape of lumbar lordosis, current knowledge indicates that overall mobility in flexion/extension decreases with advancing age [29]. Knowledge of local changes in microarchitecture and spinal function with advancing age may help to optimize treatment and it to the level of the diseased spinal segment.

The specificity of the cervical spine is highlighted by the results of the present investigation. Due to their denser trabecular structure and greater mineralization, cervical vertebrae have a significantly greater load-bearing capacity per unit area than thoracic or lumbar vertebrae.

As the sample size of the present study was rather small for multiple regression analysis, the pattern noted here will have to be validated in larger studies.

The contribution of cortical bone to the structural strength of the vertebrae and vertebral fractures remains a subject of great interest. Cortical bone has been reported to influence the prevention of insufficiency fractures [8]. Potential differences among the cervical, thoracic, and lumbar vertebrae are currently being investigated in further studies.

### Discussion of the method

The timing of the anatomical preparation did not correspond to the actual testing. Consequently, we were challenged with finding a method for the longer-term structural preservation of the samples without the strong described effects of protein denaturation and cross-linking. However, for hygienic and safety reasons, the chemical–biocidal killing of possible germs and (pathogenic) microorganisms was necessary and metabolic processes had to be stopped: additionally, we wanted to prevent dehydration. Storage in ethanol did not change the stiffness of the spines and only slightly changed their viscoelastic properties [30].

In selecting the puncture needle, we adhered to the data reported by Uhl et al. [31] who concluded, after completion of their study, that 8GJamshidi™ needles are easy to use and provide high-quality histological specimens. Furthermore, biopsies performed in animal experiments showed that a larger trepanation sample is useful for the quantitative evaluation of trabecular bone volume, trabecular density, and the number of trabeculae [32]. In the clinical setting, the central ventral approach proved to be the simplest access for obtaining biopsy samples.

For biomechanical testing, the vertebrae were prepared with a preserved vertebral arch, spinous process, and articular process. The individual vertebral bodies were isolated in advance. The intervertebral disks and the anterior and posterior longitudinal ligaments were removed.

Force was applied through oblique circular plates made of acrylic glass. Several circular plates were prepared with a band saw for angles ranging between 0° and 10° and were kept in a rough-sawn state. This caused greater friction between the vertebrae and the circular plates and prevented lateral slippage of the vertebrae. At times, we added layers of absorbent paper to increase friction. In the region of the circular plates, the estimated loss in the direction of force was 1–2%. Rotation of the circular plates achieved balance in several planes and a uniform distribution of physical force. After disinfection, the circular plates were reused in further experiments.

### Limitations

The limitations of the present study are worthy of note. The loading regimen was uniaxial uniform compression. Most vertebral fractures due to osteoporosis are caused by combined compression and rotation loads. Activities that require forward motion of the torso may place a tenfold higher compressive force on the vertebrae than activities performed when standing upright [33].

The present study is a comparative, descriptive study in which the case numbers were based on the available material. Complex statistical methods can only be applied to a limited extent. This undoubtedly limits the clinical applicability of the results.

We examined only bodies from donors with an older age; statements regarding the bone structure of younger donated bodies are therefore not possible. In addition, information on donors' medical history—especially on the type and duration of drug or physical treatments for osteoporosis—was extremely sparse. We also note the potential reinforcement effects of specimen preparation on biomechanical strength.

## Conclusions

- Because of their denser trabecular structure and greater mineral content, cervical vertebrae possess a significantly greater load-bearing capacity per unit area than thoracic or lumbar vertebrae.
- The microarchitecture of cervical vertebrae renders them less prone to fractures even in the presence of osteoporosis.
- The load-bearing capacity per unit area does not differ between men and women.
- Tb.Pf is a suitable predictor of failure load.
- The microarchitecture deteriorates after the first spinal fracture.
- Individuals with ≥ 2 VFx have more vulnerable microarchitecture.
- Due to their unique microarchitecture, cervical vertebrae do not fracture, even in osteoporosis.
- FS was significantly reduced in subjects with ≥ 2 VFx.

## Data Availability

The datasets used and/or analyzed in the current study are available from the corresponding author on reasonable request.

## Abbreviations

BMD: Bone mineral density
BMI: Body Mass Index
BVF: Bone volume fraction
C: Cervical vertebral body
cm: Centimeters
Conn.D: Connectivity density
CS: Cervical spine
CT: Computed tomography
DA: Degree of anisotropy
Fig.: Figure
FL: Failure load
FS: Failure strength
HU: Hounsfield units
kg: Kilogram
kg/m^2^: Kilograms/square meter
kV: Kilovolt
LS: Lumbar spine
L: Lumbar vertebral body
M: Mean value
mg/cm^3^: Milligrams/cubic centimeter
micro-CT: Microcomputed tomography
mm: Millimeters
mm/min: Millimeters/minute
N: Newton
N/mm^2^: Newton/square millimeter
QCT: Quantitative computed tomography
ROI: Range of interest
SD: Standard deviation
T: Thoracic vertebral body
Tab.: Table
Tb.N: Trabecular number
Tb.Pf: Trabecular pattern factor
TS: Thoracic spine
Tb.Sp: Trabecular separation
Tb.Th: Trabecular thickness
TV: Thoracic vertebrae
TX: Texas
µm: Micrometers
µA: Microamperes
VFx: Vertebral fractures
